# Four new species and three new records of helicosporous hyphomycetes from China and their multi-gene phylogenies

**DOI:** 10.3389/fmicb.2022.1053849

**Published:** 2022-11-25

**Authors:** Yong-Zhong Lu, Jian Ma, Xing-Juan Xiao, Li-Juan Zhang, Yuan-Pin Xiao, Ji-Chuan Kang

**Affiliations:** ^1^Engineering and Research Center for Southwest Bio-Pharmaceutical Resources of National Education Ministry of China, Guizhou University, Guiyang, China; ^2^School of Pharmaceutical Engineering, Guizhou Institute of Technology, Guiyang, China; ^3^Center of Excellence in Fungal Research, Mae Fah Luang University, Chiang Rai, Thailand; ^4^Guizhou Key Laboratory of Agricultural Biotechnology, Guizhou Academy of Agricultural Sciences, Guiyang, China

**Keywords:** freshwater fungi, taxonomy, Tubeufiales, woody substrates, saprophytic fungi

## Abstract

Helicosporous hyphomycetes have the potential to produce a variety of bioactive compounds. However, the strain resources of this fungal group are relatively scarce, which limits their further exploitation and utilization. In this study, based on phylogenetic analyses of combined ITS, LSU, RPB2, and TEF1α sequence data and the morphology from 11 isolates, we introduce four new species of helicosporous hyphomycetes, *viz. Helicoma wuzhishanense, Helicosporium hainanense, H. viridisporum*, and *Neohelicomyces hainanensis*, as well as three new records, *viz. Helicoma guttulatum, H. longisporum*, and *Helicosporium sexuale*. Detailed morphological comparisons of the four new species that distinguish them are provided.

## Introduction

The most remarkable feature that distinguishes helicosporous hyphomycetes from other fungal groups is that its conidia curve through at least 180° in one plane as they extend in length (Goos, 1986; Zhao et al., 2007; Luo et al., 2017; Lu et al., 2018a,b; Tian et al., 2022). They are distributed in the Dothideomycetes (Capnodiales, Microthyriales, Pleosporales, Tubeufiales, and Venturiales), Leotiomycetes (Helotiales), Orbiliomycetes (Orbiliales), Sordariomycetes (Hypocreales, Lulworthiales, Microascales, Torpedosporales), Agaricomycetes (Agaricales), Atractiellomycetes (Atractiellales), Exobasidiomycetes (Exobasidiales), Tremellomycetes (Tremellales), and Zoopagomycetes (Zoopagales) (Lu and Kang, 2020). Helicosporous fungi are widespread in tropical and temperate regions (Lu et al., 2018b). Most species in this group, which were published more than 10 years ago, were saprophytic on terrestrial woody substrates, and most of them were lacking in DNA molecular data (Goos, 1986; Zhao et al., 2007; Boonmee et al., 2014; Lu et al., 2018b). However, the species of this group discovered in the last decade mainly come from aquatic habitats (Lu et al., 2018b; Boonmee et al., 2021; Tian et al., 2022), and almost all newly published helicosporous species have molecular data. The latest comprehensive revision on helicosporous hyphomycetes was carried out by Lu et al. (2018b), who established nine new helicosporous genera based on morphology and phylogeny, *viz*. *Dematiohelicoma, Dematiohelicomyces, Dematiohelicosporum, Helicoarctatus, Helicohyalinum, Helicotruncatum, Pleurohelicosporium, Pseudohelicomyces*, and *Pseudohelicoon*, and reassessed the taxonomic system of the three earliest described helicosporous hyphomycete genera, *viz*. *Helicomyces, Helicosporium*, and *Helicoma*. For example, in the genus *Helicosporium*, Lu et al. (2018b) redefined its generic concept based on morphological and phylogenetic evidence, and accepted 13 species, including five new species, and excluded 25 species from this genus which were transferred to the genera *Neohelicosporium* and *Helicoma*. In addition, although Lu et al. (2018b) proposed some suggestions on how to classify and identify helicosporous fungi, there are still some species in this group that need more morphological and molecular data to solve their taxonomic status.

The focus of research on helicosporous fungi has been mainly in the field of taxonomy. However, these fungi are not only morphologically fascinating but also a potential source to produce a variety of bioactive secondary metabolites. For example, species of *Helicomyces, Helicosporium*, and *Helicoma* have been reported to produce natural products with antibacterial, anticancer, and anti-diabetic activities (Itazaki et al., 1990; Hanada et al., 1996; Ohtsu et al., 2003; Yoshimura et al., 2003; Zenkoh et al., 2003; Dong et al., 2004; Hu et al., 2006; Jiao et al., 2006; Jung et al., 2012; Lee et al., 2013). Furthermore, recent studies have revealed that other helicosporous fungi also show great potential for exploring new active natural products (Qian et al., 2022; Zeng et al., 2022; Zheng et al., 2022). Zheng et al. (2022) reported two novel compounds in *Tubeufia rubra*; one of which reverses multidrug resistance of tumor cell lines to Doxorubicin. Qian et al. (2022) also discovered another two new compounds in *Tubeufia rubra*, and one, namely, Rubrosin-D displayed significant multidrug resistance reversal effects. Zheng et al. (2022) discovered that some alkaloids in *Neohelicomyces hyalosporus* were cytotoxic against human cancer (A549, TCA, and RD) cells.

In order to solve the classification problems related to helicosporous hyphomycetes and enrich the species resources of the fungal group, we have recently collected a large number of specimens of this group from various terrestrial and aquatic environments. In this study, we report on 11 helicosporous hyphomycetes collected from decaying woody substrates from freshwater streams and terrestrial habitats in southern China. The taxa are characterized based on morphological features and phylogenetic analyses. The new species are morphologically and phylogenetically distinct. Detailed descriptions, illustrations, and phylogenetic analyses are provided.

## Materials and methods

### Sample collection and specimen examination

**Figure 1:**
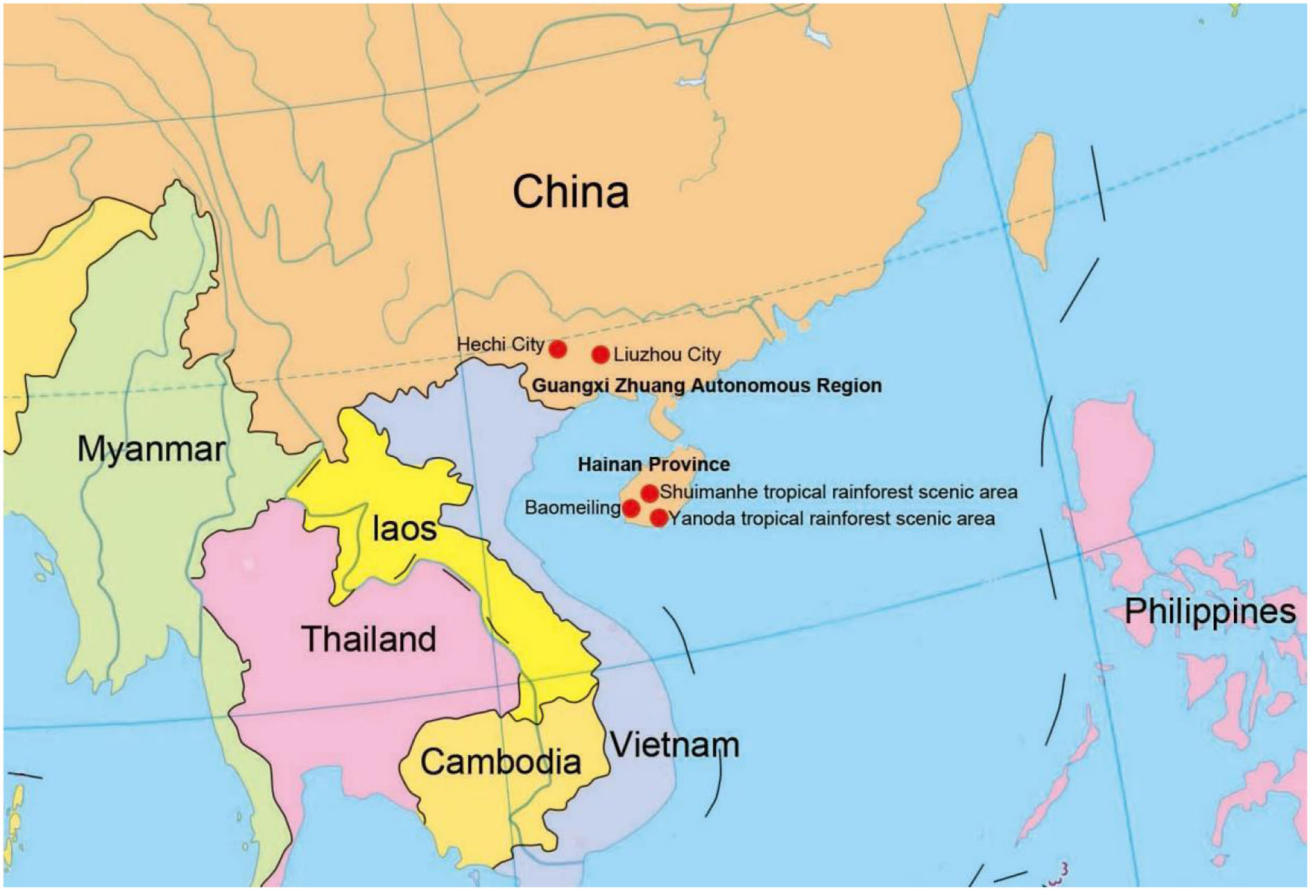
Collecting sites in this study (red dots).

Submerged decaying wood samples were collected from various sites in freshwater streams and terrestrial environments in Guangxi Zhuang Autonomous Region and Hainan Provinces, China (Figure 1). Techniques in Senanayake et al. (2020) were followed for morphological study and single spore isolation. Morphological characteristics were examined with a stereomicroscope (SMZ 745 Nikon, Japan). Micro-morphological characters were photographed using a Nikon EOS 70D digital camera attached to an ECLIPSE Ni compound microscope (Nikon, Japan). Measurements were made with a Tarosoft (R) Image Frame Work program. Figures were processed and combined using Adobe Photoshop CS6 Extended version 10.0 software (Adobe Systems, USA).

Herbarium specimens were deposited in the Herbarium of Guizhou Academy of Agriculture Sciences (Herb. GZAAS) and the Herbarium of Cryptogams Kunming Institute of Botany Academia Sinica (Herb. HKAS). Ex-type living cultures are deposited at Guizhou Culture Collection (GZCC). Facesoffungi database and Index Fungorum numbers are provided (Jayasiri et al., 2015).

### DNA extraction, PCR amplification, and sequencing

Genomic DNA was extracted from at least 3-week-old living pure cultures grown on PDA at 28 °C using the Biospin Fungus Genomic DNA Extraction Kit (BioFlux, China), and following the manufacturer's protocol. The primer pairs of ITS5/ITS4, LR0R/LR5, fRPB2-5F/fRPB2-7cR, and EF1-983F/EF1-2218R were used to amplify the internal transcribed spacer (ITS) (White et al., 1990), the large subunit ribosomal DNA (LSU) (Vilgalys and Hester, 1990), the RNA polymerase II second largest subunit (RPB2) (Liu et al., 1999), and the translation elongation factor 1-alpha gene (TEF1α) (Rehner and Buckley, 2005) regions, respectively. The ITS, LSU, RPB2, and TEF1α amplification reactions were carried out using the method described by Lu et al. (2017b, 2018a). The PCR products were purified and sequenced with the same primers at Tsingke Biological Technology (Kunming) Co., China.

### Phylogenetic analysis

DNASTAR Lasergene SeqMan Pro v. 7.1.0 (44.1) was used to edit ambiguous bases at both ends of the raw forward and reverse reads and to assemble them. The newly obtained sequences were used as queries to perform BLAST searches against the nr database to check for contamination, compare species, and create datasets. MAFFT v.7 was used to align the individual datasets (Katoh et al., 2019). Each alignment was trimmed using Trimal (Capella-Gutiérrez et al., 2009). BioEdit was used to check the alignment manually (Hall, 1999).

Four genetic markers, including ITS, LSU, RPB2, and TEF1α, were used for phylogenetic inferences (Table 1). The phylogeny tree was inferred using 147 taxa. IQ-Tree v.2 (Minh et al., 2020) was used to infer maximum likelihood trees (ML) according to the Bayesian information criterion (BIC). Partitioned analyses were carried out for the combined datasets, which were partitioned according to genetic markers. Branch support was estimated from 1,000 ultrafast bootstrap replicates. RAxML-HPC2 on XSEDE (8.2.12) (Stamatakis, 2014) in the CIPRES Science Gateway platform was also used. ModelTest, as implemented in MrMTgui (Nuin, 2007), was used to determine the best-fit evolution model for Bayesian inference analyses using the Akaike Information Criterion (AIC). Bootstrap support was estimated from 1,000 rapid bootstrap replicates. MrBayes v.3.1.2 (Ronquist et al., 2012) was utilized to evaluate the posterior probabilities (PP) by Markov Chain Monte Carlo sampling (MCMC). The number of generations was determined separately for each dataset and is noted in the individual tree legends. The first 25% of the trees were discarded, as they represented the burn-in phase of the analyses, while the remaining were used for calculating PP in the majority rule consensus tree. For all Bayesian inference trees, convergence was declared when the average standard deviation reached 0.01. The trees were figured in the FigTree v1.4.0 program (Rambaut and Drummond, 2008). The approximately unbiased (AU) test, implemented in CONSEL, was used to test the placement of the newly erected family (Shimodaira and Hasegawa, 2001). Topologies with AU test *p*-values < 0.05 were rejected.

**Table 1:** Taxa used in this study and their GenBank accession numbers for ITS, LSU, RPB2, and TEF1α DNA sequence data.

**Taxa**	**Strain/Voucher No.^b^**	**GenBank accession no**.			
		**ITS**	**LSU**	**TEF1α**	**RPB2**
*Acanthohelicospora aurea*	GZCC 16-0060	KY321323	KY321326	KY792600	MF589911
*Acanthohelicospora pinicola*	MFLUCC 10-0116	KF301526	KF301534	KF301555	–^a^
*Acanthostigma chiangmaiensis*	MFLUCC 10-0125	JN865209	JN865197	KF301560	–
*Acanthostigma perpusillum*	UAMH 7237	AY916492	AY856892	–	–
*Acanthostigmina multiseptatum*	ANM 475	GQ856145	GQ850492	–	–
*Acanthostigmina multiseptatum*	ANM 665	GQ856144	GQ850493	–	–
*Aquaphila albicans*	BCC 3543	DQ341096	DQ341101	–	–
*Aquaphila albicans*	MFLUCC 16-0010	KX454165	KX454166	KY117034	MF535255
*Berkleasmium fusiforme*	MFLUCC 17-1978	MH558693	MH558820	MH550884	MH551007
*Berkleasmium longisporum*	MFLUCC 17-1999	MH558698	MH558825	MH550889	MH551012
*Boerlagiomyces macrospora*	MFLUCC 12-0388	KU144927	KU764712	KU872750	–
*Botryosphaeria agaves*	MFLUCC 10-0051	JX646790	JX646807	–	–
*Botryosphaeria dothidea*	CBS 115476	KF766151	DQ678051	DQ767637	DQ677944
*Chlamydotubeufia cylindrica*	MFLUCC 16-1130	MH558702	MH558830	MH550893	MH551018
*Chlamydotubeufia huaikangplaensis*	MFLUCC 10-0926	JN865210	JN865198	–	–
*Chlamydotubeufia krabiensis*	MFLUCC 16-1134	KY678767	KY678759	KY792598	MF535261
*Dematiohelicoma pulchrum*	MUCL 39827	AY916457	AY856872	–	–
*Dematiohelicomyces helicosporus*	MFLUCC 16-0003	KX454169	KX454170	KY117035	MF535258
*Dematiohelicomyces helicosporus*	MFLUCC 16-0007	MH558703	MH558831	MH550894	MH551019
*Dematiohelicomyces helicosporus*	MFLUCC 16-0213	KX454169	KX454170	KY117035	MF535258
*Dematiohelicosporum guttulatum*	MFLUCC 17-2011	MH558705	MH558833	MH550896	MH551021
*Dematiotubeufia chiangraiensis*	MFLUCC 10-0115	JN865200	JN865188	KF301551	–
*Dictyospora thailandica*	MFLUCC 16-0001	KY873627	KY873622	KY873286	–
*Dictyospora thailandica*	MFLUCC 16-0215	KY873628	KY873623	KY873287	–
*Helicangiospora lignicola*	MFLUCC 11-0378	KF301523	KF301531	KF301552	–
*Helicoarctatus aquaticus*	MFLUCC 17-1996	MH558707	MH558835	MH550898	MH551024
*Helicodochium aquaticum*	MFLUCC 17-2016	MH558709	MH558837	MH550900	MH551026
*Helicodochium aquaticum*	MFLUCC 18-0490	MH558710	MH558838	MH550901	MH551027
*Helicohyalinum aquaticum*	MFLUCC 16-1131	KY873625	KY873620	KY873284	MF535257
*Helicohyalinum infundibulum*	MFLUCC 16-1133	MH558712	MH558840	MH550903	MH551029
*Helicoma ambiens*	UAMH 10533	AY916451	AY856916	–	–
*Helicoma ambiens*	UAMH 10534	AY916450	AY856869	–	–
*Helicoma aquaticum*	MFLUCC 17-2025	MH558713	MH558841	MH550904	MH551030
*Helicoma brunneisporum*	MFLUCC 17-1983	MH558714	MH558842	MH550905	MH551031
*Helicoma dennisii*	NBRC 30667	AY916455	AY856897	–	–
*Helicoma freycinetiae*	MFLUCC 16-0363	MH275062	MH260295	MH412770	–
*Helicoma fusiforme*	MFLUCC 17-1981	MH558715	–	MH550906	–
***Helicoma guttulatum***	**GZCC 22-2004**	**OP508739**	**OP508779**	**OP698090**	**OP698079**
***Helicoma guttulatum***	**GZCC 22-2024**	**OP508733**	**OP508773**	**OP698084**	**OP698073**
***Helicoma guttulatum***	**GZCC 22-2025**	**OP508737**	**OP508777**	**OP698088**	**OP698077**
*Helicoma guttulatum*	MFLUCC 16-0022	KX454171	KX454172	MF535254	–
*Helicoma guttulatum*	MFLUCC 21-0152	OL545456	OL606150	OL964521	OL964527
***Helicoma wuzhishanense***	**GZCC 22-2003**	**OP508732**	**OP508772**	**OP698083**	**OP698072**
*Helicoma hongkongense*	MFLUCC 17-2005	MH558716	MH558843	MH550907	MH551033
*Helicoma hydei*	MFLUCC 18-1270	MH747116	MH747101	MH747100	–
*Helicoma inthanonense*	MFLUCC 11-0003	JN865211	JN865199	–	–
*Helicoma khunkornensis*	MFLUCC 10-0119	JN865203	JN865191	KF301559	–
*Helicoma linderi*	NBRC 9207	AY916454	AY856895	–	–
***Helicoma longisporum***	**GZCC 22-2005**	**OP508740**	**OP508780**	**OP698091**	**OP698080**
***Helicoma longisporum***	**GZCC 22-2026**	**OP508738**	**OP508778**	**OP698089**	**OP698078**
*Helicoma longisporum*	MFLUCC 16-0002	MH558717	MH558844	MH550908	MH551034
*Helicoma longisporum*	MFLUCC 16-0005	MH558718	–	MH550909	MH551035
*Helicoma longisporum*	MFLUCC 16-0211	MH558719	MH558845	MH550910	MH551036
*Helicoma longisporum*	MFLUCC 17-1997	MH558720	MH558846	MH550911	MH551037
*Helicoma miscanthi*	MFLUCC 11-0375	KF301525	KF301533	KF301554	–
*Helicoma muelleri*	CBS 964.69	AY916453	AY856877	–	–
*Helicoma muelleri*	UBC F13877	AY916452	AY856917	–	–
*Helicoma multiseptatum*	GZCC 16-0080	MH558721	MH558847	MH550912	MH551038
*Helicoma nematosporum*	MFLUCC 16-0011	MH558722	MH558848	MH550913	MH551039
*Helicoma rubriappendiculatum*	MFLUCC 18-0491	MH558723	MH558849	MH550914	MH551040
*Helicoma rufum*	MFLUCC 17-1806	MH558724	MH558850	MH550915	–
*Helicoma rugosum*	ANM 1169	–	GQ850484	–	–
*Helicoma rugosum*	ANM 196	GQ856138	GQ850482	–	–
*Helicoma rugosum*	JCM 2739	–	AY856888	–	–
*Helicoma septoconstrictum*	MFLUCC 17-1991	MH558725	MH558851	MH550916	MH551041
*Helicoma septoconstrictum*	MFLUCC 17-2001	MH558726	MH558852	MH550917	MH551042
*Helicoma siamense*	MFLUCC 10-0120	JN865204	JN865192	KF301558	–
*Helicoma* sp.	HKUCC 9118	–	AY849966	–	–
*Helicoma tectonae*	MFLUCC 12–0563	KU144928	KU764713	KU872751	–
*Helicomyces chiayiensis*	BCRC FU30842	LC316604	–	–	–
*Helicomyces hyalosporus*	MFLUCC 17–0051	MH558731	MH558857	MH550922	MH551047
*Helicomyces torquatus*	MFLUCC 16–0217	MH558732	MH558858	MH550923	MH551048
*Helicosporium aquaticum*	MFLUCC 17-2008	MH558733	MH558859	MH550924	MH551049
*Helicosporium flavisporum*	MFLUCC 17-2020	MH558734	MH558860	MH550925	MH551050
*Helicosporium flavum*	MFLUCC 16-1230	KY873626	KY873621	KY873285	–
***Helicosporium hainanense***	**GZCC 22-2006**	**OP508730**	**OP508770**	**OP698081**	**OP698070**
*Helicosporium luteosporum*	MFLUCC 16-0226	KY321324	KY321327	KY792601	–
*Helicosporium luteosporum*	MFLUCC 16-1233	–	KY873624	–	–
*Helicosporium setiferum*	BCC 3332	AY916490	AY856907	–	–
*Helicosporium setiferum*	BCC 8125	AY916491	–	–	–
*Helicosporium setiferum*	MFLUCC 17-1994	MH558735	MH558861	MH550926	MH551051
*Helicosporium setiferum*	MFLUCC 17-2006	MH558736	MH558862	MH550927	MH551052
*Helicosporium setiferum*	MFLUCC 17-2007	MH558737	MH558863	MH550928	MH551053
***Helicosporium sexuale***	**GZCC 22-2007**	**OP508731**	**OP508771**	**OP698082**	**OP698071**
*Helicosporium sexuale*	MFLUCC 16-1244	MZ538503	MZ538537	MZ567082	MZ567111
*Helicosporium sp*.	NBRC 9014	AY916489	AY856903	–	–
*Helicosporium vegetum*	CBS 254.75	–	DQ470982	DQ471105	–
*Helicosporium vegetum*	CBS 269.52	AY916487	AY856893	–	–
*Helicosporium vegetum*	CBS 941.72	AY916488	AY856883	–	–
*Helicosporium vegetum*	NBRC 30345	–	AY856896	–	–
*Helicosporium vesicarium*	MFLUCC 17-1795	MH558739	MH558864	MH550930	MH551055
*Helicosporium viridiflavum*	MFLUCC 17-2336	MH558738	–	MH550929	MH551054
***Helicosporium viridisporum***	**GZCC 22-2008**	**OP508736**	**OP508776**	**OP698087**	**OP698076**
*Helicotruncatum palmigenum*	KUMCC 21-0474	OM102542	OL985959	OM355488	OM355492
*Helicotruncatum palmigenum*	NBRC 32663	AY916480	AY856898	–	–
*Helicotubeufia guangxiensis*	MFLUCC 17-0040	MH290018	MH290023	MH290028	MH290033
*Helicotubeufia jonesii*	MFLUCC 17-0043	MH290020	MH290025	MH290030	MH290035
*Kevinhydea brevistipitata*	MFLUCC 18-1269	MH747115	MH747102	–	–
*Manoharachariella tectonae*	MFLUCC 12-0170	KU144935	KU764705	KU872762	–
*Muripulchra aquatica*	KUMCC 15-0276	KY320534	KY320551	KY320564	–
*Muripulchra aquatica*	MFLUCC 15-0249	KY320532	KY320549	–	–
*Neoacanthostigma fusiforme*	MFLUCC 11-0510	KF301529	KF301537	–	–
*Neochlamydotubeufia fusiformis*	MFLUCC 16–0016	MH558740	MH558865	MH550931	MH551059
*Neochlamydotubeufia khunkornensis*	MFLUCC 10–0118	JN865202	JN865190	KF301564	–
*Neohelicoma fagacearum*	MFLUCC 11-0379	KF301524	KF301532	KF301553	–
*Neohelicomyces aquaticus*	KUMCC 15-0463	KY320529	KY320546	KY320562	–
*Neohelicomyces aquaticus*	KUNCC 21-10703	–	MZ841660	–	–
*Neohelicomyces aquaticus*	MFLUCC 16-0993	KY320528	KY320545	KY320561	–
*Neohelicomyces grandisporus*	KUMCC 15-0470	KX454173	KX454174	–	MH551067
***Neohelicomyces hainanensis***	**GZCC 22-2009**	**OP508734**	**OP508774**	**OP698085**	**OP698074**
***Neohelicomyces hainanensis***	**GZCC 22-2027**	**OP508735**	**OP508775**	**OP698086**	**OP698075**
*Neohelicomyces hyalosporus*	GZCC 16-0086	MH558745	MH558870	MH550936	MH551064
*Neohelicomyces longisetosus*	NCYU 106H1-1-1	MT939303	–	–	–
*Neohelicomyces pallidus*	CBS 245.49	**–**	GU566745	–	–
*Neohelicomyces pallidus*	CBS 271.52	AY916461	AY856887	–	–
*Neohelicomyces pallidus*	CBS 962.69	AY916460	AY856886	–	–
*Neohelicomyces pallidus*	UAMH 10535	AY916462	AY856913	–	–
*Neohelicomyces pandanicola*	KUMCC 16-0143	NR_168180	MH260307	MH41277	–
*Neohelicomyces submersus*	MFLUCC 16-1106	KY320530	KY320547	–	–
*Neohelicosporium aquaticum*	MFLUCC 17-1519	MF467916	MF467929	MF535242	MF535272
*Neohelicosporium astrictum*	MFLUCC 17-2004	MH558747	MH558872	MH550938	MH551070
*Neohelicosporium ellipsoideum*	MFLUCC 16-0229	MH558748	MH558873	MH550939	MH551071
*Neohelicosporium guangxiense*	MFLUCC 17-1522	MF467922	MF467935	MF535248	MF535278
*Neohelicosporium hyalosporum*	GZCC 16-0076	MF467923	MF467936	MF535249	MF535279
*Neohelicosporium irregulare*	MFLUCC 17-1796	MH558752	MH558877	MH550943	MH551075
*Neohelicosporium krabiense*	MFLUCC 16-0224	MH558754	MH558879	MH550945	MH551077
*Neohelicosporium laxisporum*	MFLUCC 17-2027	MH558755	MH558880	MH550946	MH551078
*Neohelicosporium ovoideum*	GZCC 16-0064	MH558756	MH558881	MH550947	MH551079
*Neohelicosporium parvisporum*	MFLUCC 17-1523	MF467926	MF467939	MF535252	MF535282
*Neohelicosporium thailandicum*	MFLUCC 16-0221	MF467928	MF467941	MF535253	MF535283
*Neotubeufia krabiensis*	MFLUCC 16-1125	MG012031	MG012024	MG012010	MG012017
*Parahelicomyces aquaticus*	MFLUCC 16-0234	MH558766	MH558891	MH550958	MH551092
*Parahelicomyces chiangmaiensis*	MFLUCC 21-0159	OL697884	OL606145	OL964516	OL964522
*Parahelicomyces talbotii*	MFLUCC 17-2021	MH558765	MH558890	MH550957	MH551091
*Parahelicomyces yunnanensis*	CGMCC 3.20429	MZ092717	MZ841658	–	OM022000
*Pleurohelicosporium parvisporum*	MFLUCC 17-1982	MH558764	MH558889	MH550956	MH551088
*Pseudohelicoon gigantisporum*	BCC 3550	AY916467	AY856904	–	–
*Pseudohelicoon subglobosum*	NCYU K3-2-3	LC316609	LC316612	–	–
*Tamhinispora indica*	NFCCI 2924	KC469282	KC469283	–	–
*Tamhinispora srinivasanii*	NFCCI 4231	MG763746	MG763745	–	–
*Thaxteriellopsis lignicola*	MFLUCC 16-0026	MH558768	MH558893	MH550960	MH551094
*Thaxteriellopsis lignicola*	MFLUCC 10-0124	JN865208	JN865196	KF301561	–
*Tubeufia bambusicola*	MFLUCC 17-1803	MH558771	MH558896	MH550963	MH551097
*Tubeufia brevis*	MFLUCC 17-1799	MH558772	MH558897	MH550964	MH551098
*Tubeufia javanica*	MFLUCC 12-0545	KJ880034	KJ880036	KJ880037	–
*Tubeufia rubra*	GZCC 16-0081	MH558801	MH558926	MH550994	MH551128

New sequences are in bold.

a No data in GenBank.

b ANM, A.N. Miller; BBB, Bahía Blanca Biology Herbarium, Argentina; BCC, BIOTEC Culture Collection, Thailand; CBS, Centra albureau voor Schimmel cultures, Utrecht, The Netherlands; CGMCC, the China General Microbiological Culture Collection Center, Beijing, China; GZCC, Guizhou Culture Collection, Guizhou Academy of Agricultural Sciences, Guiyang, China; JCM, Japan Collection of Microorganisms; KUMCC, Culture collection of Kunming Institute of Botany, Kunming, China; MFLU, the Herbarium of Mae Fah Luang University; MFLUCC, Mae Fah Luang University Culture Collection, Chiang Rai, Thailand; MUCL, Mycothèque de l'Université Catholique de Louvain, Louvain-la-Neuve, Belgium; NBRC, the NITE Biological Resource Center; NCYU, National Chiayi University, Taiwan, China; NFCCI, the National Fungal Culture Collection of India; UAMH, UAMH Center for Global Microfungal Biodiversity, University of Toronto, Canada; UBC, University of British Columbia, Canada.

## Results

### Phylogenetic analysis of combined ITS, LSU, RPB2, and TEF1α sequence data

**Figure 2:**
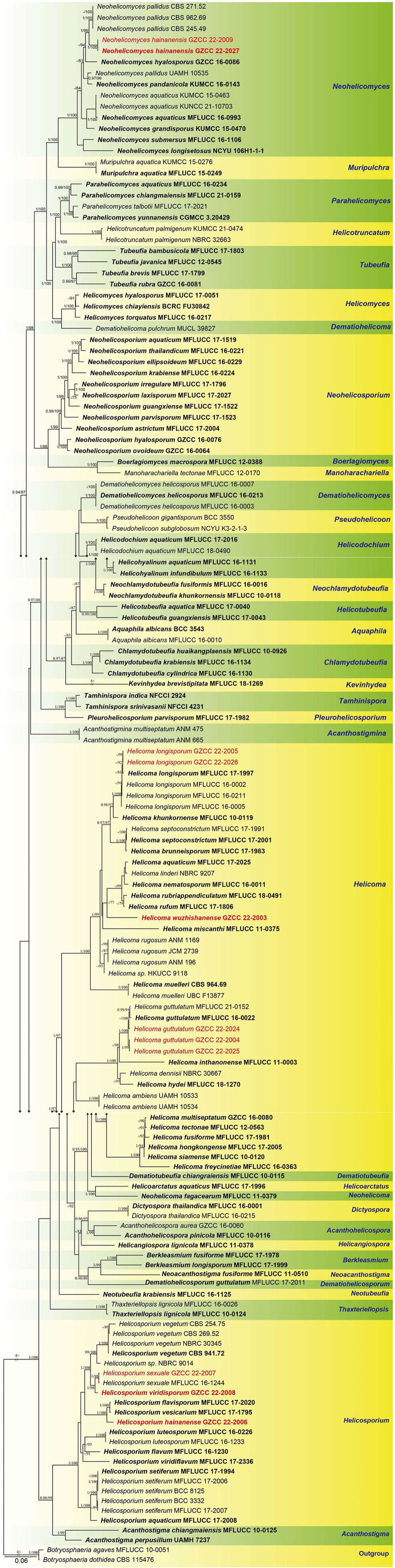
Phylogenetic tree generated from a maximum likelihood analysis based on a concatenated alignment of ITS, LSU, RPB2, and TEF1α sequence data. Bootstrap support values of maximum likelihood (ML) ≥75% and Bayesian posterior probabilities (PP) ≥0.95 are given near the nodes as PP/MLBS. The tree is rooted with *Botryosphaeria agaves* (MFLUCC 10-0051) and *B. dothidea* (CBS 115,476). Newly generated sequences are in red. Ex-type strains are in bold.

The combined ITS, LSU, RPB2, and TEF1α datasets comprised 11 newly sequenced strains. Multiple genes were concatenated, which comprised 146 taxa and 3313 nucleotide characters, including gaps (ITS: 513 bp; LSU: 843 bp; RPB2: 1045 bp; TEF1α: 912 bp). The maximum likelihood and Bayesian analysis of the combined dataset resulted in phylogenetic reconstructions with largely similar topologies, and the IQ-Tree is shown in Figure 2.

Representatives of the sequenced genera (with molecular data) of helicosporous hyphomycetes (Boonmee et al., 2011, 2014; Rajeshkumar and Sharma, 2013; Brahamanage et al., 2017; Doilom et al., 2017; Lu et al., 2017a, 2018a,b; Luo et al., 2017; Phookamsak et al., 2017; Liu et al., 2019; Tian et al., 2022) are included in our phylogenetic analysis (Figure 2). Thirty-six genera are represented by at least one species in Tubeufiaceae. Our 11 isolates are recognized as four new species, *viz. Helicoma wuzhishanense, Helicosporium hainanense, H. viridisporum*, and *Neohelicomyces hainanensis*, and three new records, *viz. Helicoma guttulatum, H. longisporum*, and *Helicosporium sexuale*.

### Taxonomy

*Helicoma guttulatum* Y.Z. Lu, Boonmee & K.D. Hyde, Fungal Diversity 80: 125 (2016), Figure 3.

**Figure 3:**
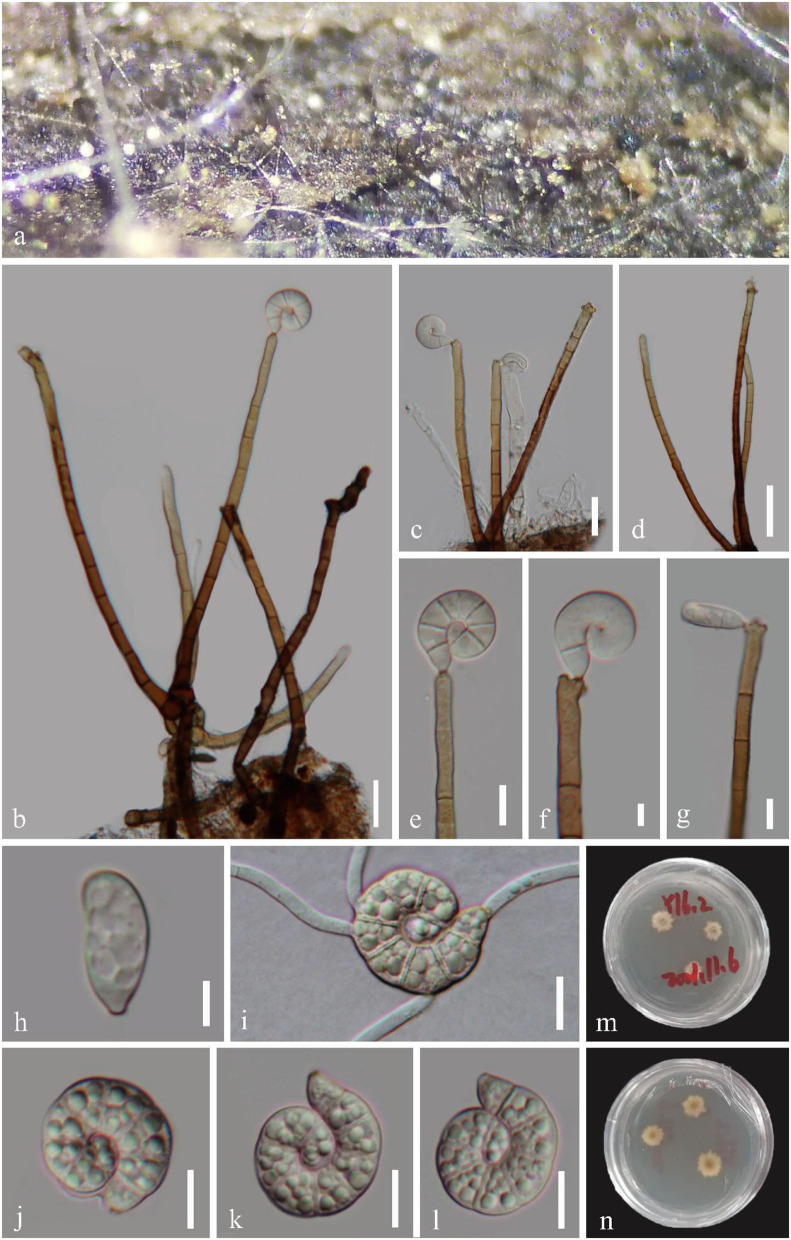
*Helicoma guttulatum* (GZAAS 22-2004). **(a)** Colony on decaying wood. **(b–d)** Conidiophores and conidia. **(e–g)** Conidiogenous cells. **(i)** Germinating conidium. **(h,j–l)** Conidia. **(m,n)** Colonies on PDA observed from above and below. Scale bars: **(b–d)** = 20 μm, **(e–j,i–l)** = 10 μm, and **(h)** = 5 μm.

*Index Fungorum number*: IF 552218; *Facesoffungi number*: FoF 02358.

*Saprobic* on submerged decaying wood in a freshwater stream. **Sexual morph** Undetermined. **Asexual morph** Hyphomycetous, helicosporous. *Colonies* superficial, effuse, gregarious, brown to dark brown. *Mycelium* mostly immersed, composed of branched, septate, brown hyphae. *Conidiophores* 120–202 × 4–6.5 μm ($\bar{x}$ = 169 × 5.5 μm, *n* = 20), macronematous, mononematous, cylindrical, erect, septate, unbranched, pale brown to brown at the apex, dark brown at the base, smooth-walled. *Conidiogenous cells* 18–37 × 4.5–6 μm ($\bar{x}$ = 24 × 5 μm, *n* = 20), holoblastic, mono- to polyblastic, integrated, terminal, cylindrical, brown, and smooth-walled. *Conidia* 20–26.5 μm ($\bar{x}$ = 22 μm, *n* = 25) in diam., and conidial filament 7.5–9.5 μm ($\bar{x}$ = 8.5 μm, *n* = 25) wide and 43–57 μm long ($\bar{x}$ = 51.5 μm, *n* = 25), solitary, acrogenous, helicoid, tightly coiled 1–1^1^/_2_ times, guttulate, do not become loose in water, 7–8-septate, straight constricted at the septa, subhyaline to pale brown, tapering toward the flat end, rounded at the apex, conico-truncate at the base, smooth-walled.

*Culture characteristics*: *Conidia* germinating on PDA within 12 h; *Colonies* growing on PDA, reaching 9 mm in 2 weeks at 25°C, circular, with a flat surface, edge undulate, and pale brown to brown in the PDA medium.

*Material examined*: CHINA, Hainan Province, Yanoda Tropical rainforest scenic area, on submerged decaying wood in a freshwater stream, 23 October 2021, Jian Ma, Y16.2 (GZAAS 22-2004), living culture, GZCC 22-2004; Ibid., Y4 (GZAAS 22-2025), living culture, GZCC 22-2025; Hainan Province, Wuzhishan City, Shuimanhe tropical rainforest scenic area in Wuzhishan, on submerged decaying wood in a freshwater stream, 15 August 2021, Jian Ma, WZS34 (GZAAS 22-2024), living culture, GZCC 22-2024.

*GenBank accession numbers*: GZCC 22-2004: OP508739 (ITS), OP508779 (LSU), OP698079 (RPB2), and OP698090 (TEF1α); GZCC 22-2025: OP508737 (ITS), OP508777 (LSU), OP698077 (RPB2), and OP698088 (TEF1α); GZCC 22-2024: OP508733 (ITS), OP508773 (LSU), OP698073 (RPB2), and OP698084 (TEF1α).

Notes: *Helicoma guttulatum* was introduced by Hyde et al. (2016) with morphological and phylogenetic evidence. Tian et al. (2022) reported a new collection from Thailand. In this study, three newly obtained isolates clustered with two known strains of *H. guttulatum* (MFLUCC 16-0022 and MFLUCC 21-0152) with high statistical support (100% ML/1.00 PP, Figure 2). We note that there are two isolates (GZCC 22-2004 and GZCC 22-2025) clustered together with high statistical support and were phylogenetically different from the other isolates. However, there are only 5 bp and 12 bp differences in ITS and RPB2 between them and the ex-type strain of *H. guttulatum* (MFLUCC 16-0022), and their LSU and TEF1α data are identical. Moreover, we could not identify any morphological character differences to separate them, and these few gene base pair changes are within the accepted range of variation for a species; thus, we identify the newly obtained isolates as *H. guttulatum*. This species has only been previously reported in Thailand. It is the first record of *H. guttulatum* in China and in a terrestrial habitat.

***Helicoma longisporum*** Y.Z. Lu, J.K. Liu & K.D. Hyde, Fungal Diversity 92: 178 (2018), Figure 4.

**Figure 4:**
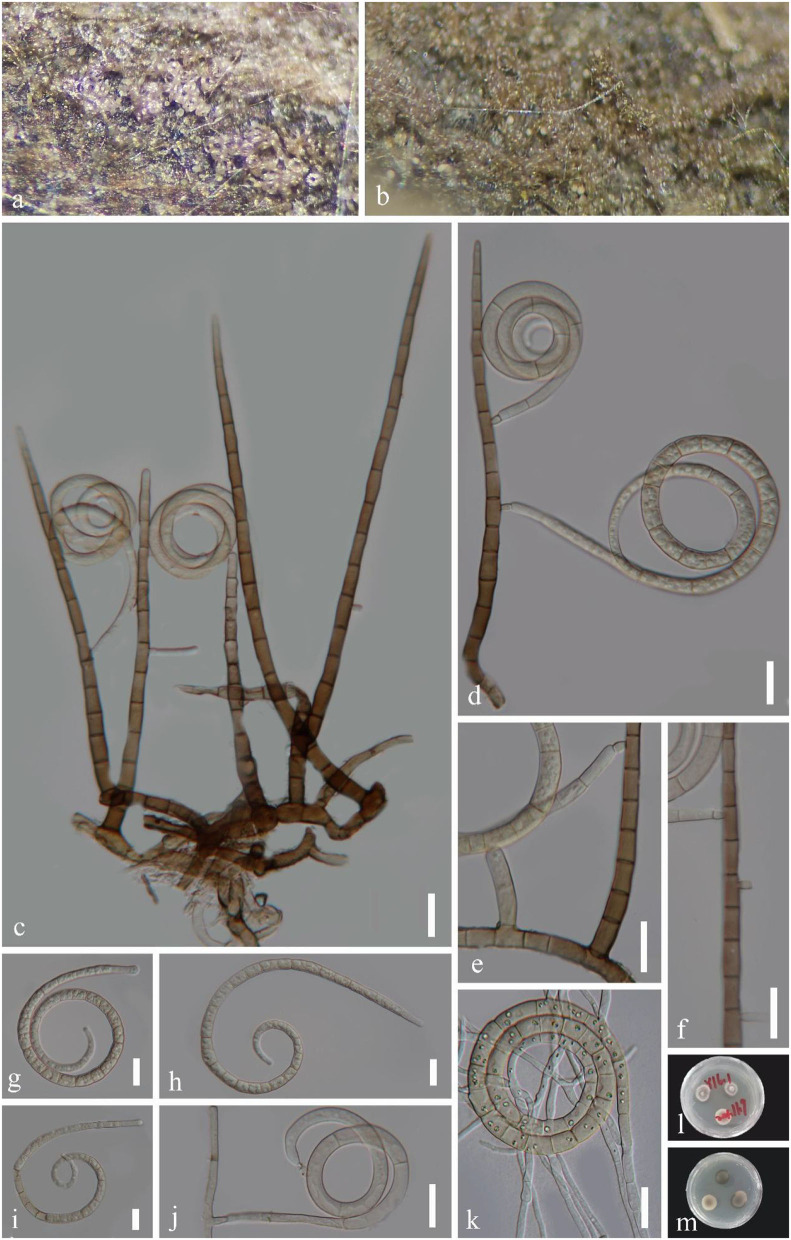
*Helicoma longisporum* (GZAAS 22-2005). **(a,b)** Colony on decaying wood. **(c,d)** Conidiophores with attached conidia. **(e,f,j)** Conidiogenous cells. **(g–i)** Conidia. **(k)** Germinating conidium. **(l,m)** Colonies on PDA observed from above and below. Scale bars: **(c–k)** = 20 μm.

*Index Fungorum number*: IF 554840; *Facesoffungi number*: FoF 04715.

*Saprobic* on decaying wood in a freshwater stream. **Sexual morph** Undetermined. **Asexual morph** Hyphomycetous, helicosporous. *Colonies* on the substratum superficial, effuse, gregarious, light pink to brown. *Mycelium* partly immersed, pale brown to brown, septate, branched hyphae, with masses of crowded, glistening conidia. *Conidiophores* 114–281 × 6–10.5 μm ($\bar{x}$ = 197.5 × 7 μm, *n* = 20), macronematous, mononematous, cylindrical, straight, unbranched, septate, pale brown to brown, smooth-walled. *Conidiogenous cells* 11–21 × 6.5–10 μm ($\bar{x}$ = 13.5 × 7.5 μm, *n* = 20), holoblastic, monoblastic, integrated, intercalary, cylindrical, with denticles, rising laterally from the lower portion of conidiophores as tiny tooth-like protrusions (3–5.5 μm long, 3.5–4.5 μm wide), pale brown, smooth-walled. *Conidia* 51–70 μm in diam. and conidial filament 6.5–11 μm wide ($\bar{x}$ = 61 × 9 μm, *n* = 20), 325–508 μm long, solitary, pleurogenous, helicoid, coiled 2–3 times, becoming loosely coiled in water, rounded at tip, up to 34-septate, constricted at septa, pale brown to brown, smooth-walled.

*Culture characteristics*: *Conidia* germinating on PDA within 12 h. *Colonies* growing on PDA, reaching 10 mm in 2 weeks at 25°C, circular, with a flat surface, edge entire, and pale brown to brown in the PDA medium.

*Material examined*: CHINA, Hainan Province, Yanoda Tropical rainforest scenic area, on submerged decaying wood in a freshwater stream, 23 October 2021, Jian Ma, Y16.3 (GZAAS 22-2005), living culture, GZCC 22-2005; Ibid., Y5 (GZAAS 22-2026), living culture, GZCC 22-2026.

*GenBank accession numbers*: GZCC 22-2005: OP508740 (ITS), OP508780 (LSU), OP698080 (RPB2), and OP698091 (TEF1α); GZCC 22-2026: OP508738 (ITS), OP508778 (LSU), OP698078 (RPB2), and OP698089 (TEF1α).

Notes: *Helicoma longisporum* was introduced by Lu et al. (2018b) based on morphology and phylogeny. In this study, two newly obtained isolates are identified as *H. longisporum* based on their identical DNA molecular data, conidiophores, conidiogenous cells, and conidial characteristics (Lu et al., 2018b). This species has only been previously reported in Thailand (Lu et al., 2018b). It is the first record of *H. longisporum* in China.

**Figure 5:**
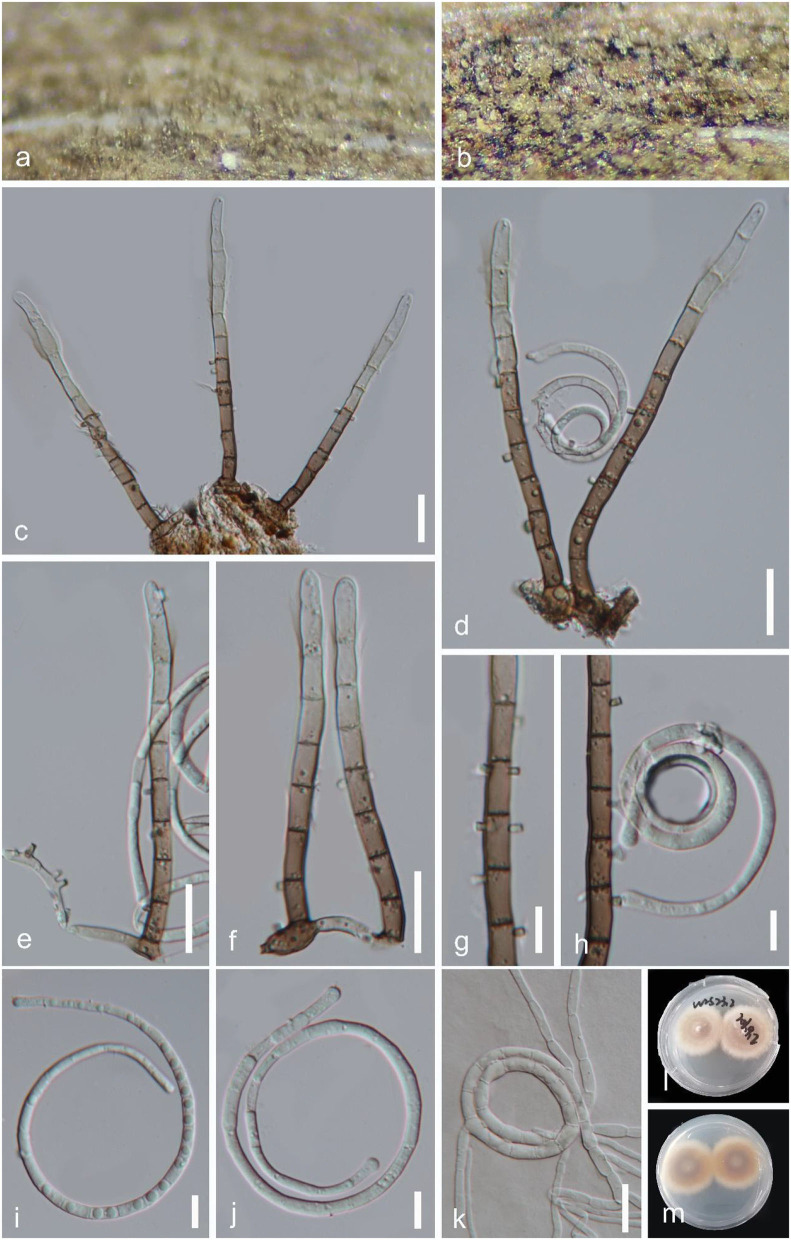
*Helicoma wuzhishanense* (GZAAS 22-2003, holotype). **(a,b)** Colony on decaying wood. **(c–f)** Conidiophores. **(g,h)** Conidiogenous cells with attached conidium. **(i,j)** Conidia. **(k)** Germinating conidium. **(l,m)** Colonies on PDA observed from above and below. Scale bars: **(c–f,k)** = 20 μm, **(g–j)** = 10 μm.

***Helicoma wuzhishanense*** Y.Z. Lu & J.C. Kang, sp. nov. Figure 5.

*Index Fungorum number*: IF 900032; *Facesoffungi number*: FoF 13100.

*Holotype*: GZAAS 22-2003.

*Etymology*: **“***wuzhishanense*” referring to collecting site.

*Saprobic* on decaying wood in a freshwater stream. **Sexual morph** Undetermined. **Asexual morph** Hyphomycetous, helicosporous. *Colonies* on the substratum superficial, effuse, gregarious, brown to dark brown. *Mycelium* partly immersed, brown, septate, branched hyphae, with masses of crowded, glistening conidia. *Conidiophores* 90–130 μm long, 5.5–6.5 μm wide ($\bar{x}$ = 115 × 6 μm, *n* = 30), macronematous, mononematous, cylindrical, erect, straight to slightly bent, unbranched, septate, the lower part brown and the upper part pale brown, smooth-walled. *Conidiogenous cells* 10–13 × 5–6.5 μm ($\bar{x}$ = 11.5 × 5.5 μm, *n* = 20), holoblastic, mono- to polyblastic, integrated, intercalary, cylindrical, with denticles, rising laterally from the lower portion of conidiophores as tiny tooth-like protrusions (1.5–3 μm long, 1.5–2.5 μm wide), brown, smooth-walled. *Conidia* 34–58 μm diam., and conidial filament 2.5–5 μm wide ($\bar{x}$ = 45 × 4 μm, *n* = 20), 182–287 μm long, up to 34-septate, solitary, pleurogenous, helicoid, coiled 2^1^/_3_-3^1^/_3_ times, becoming loosely coiled in water, rounded at tip, guttulate, hyaline to pale brown, smooth-walled.

*Culture characteristics*: *Conidia* germinating on water agar and germ tubes produced from conidia within 12 h. *Colonies* growing on PDA, circular, with a flat surface, edge entire, reaching 29 mm in 4 weeks at 25°C, pale brown to yellowish in the PDA medium.

*Material examined*: CHINA, Hainan Province, Wuzhishan City, Shuimanhe tropical rainforest scenic area in Wuzhishan, on submerged decaying wood in a freshwater stream, 15 August 2021, Jian Ma, WZS23.2 (GZAAS 22-2003, holotype; HKAS 125862, isotype), ex-type living culture, GZCC 22-2003.

*GenBank accession numbers*: OP508732 (ITS), OP508772 (LSU), OP698072 (RPB2), and OP698083 (TEF1α).

*Notes*: Morphologically, *Helicoma wuzhishanense* resembles *Helicoma rufum*, having unbranched, straight to slightly bent, cylindrical conidiophores, and pleurogenous helicoid conidia. However, *H. wuzhishanense* can be distinguished from *H*. *rufum* by its smaller conidiophores (90–130 μm × 5.5–6.5 μm *vs*. 110–210 μm × 7–8.5 μm) and shorter conidial filament (182–287 μm *vs*. 240–410 μm) (Lu et al., 2018b). Furthermore, *H. rufum* produces a reddish brown pigment in the PDA medium in 7 days but *H. wuzhishanense* lacks this characteristic. Phylogenetically, *H. wuzhishanense* formed an independent lineage within the genus (Figure 2) and the phylogenetic analysis result supports it as a distinct species.

**Figure 6:**
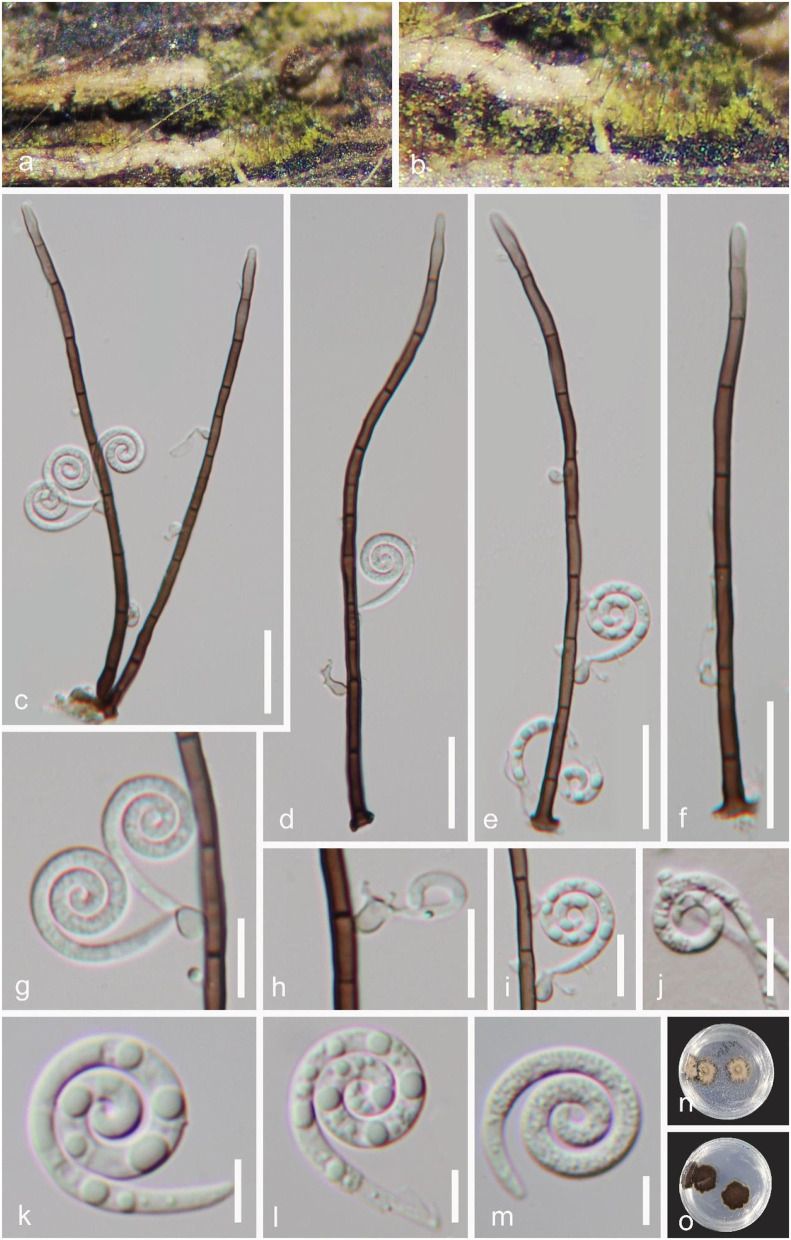
*Helicosporium hainanense* (GZAAS 22-2006, holotype). **(a,b)** Colony on decaying wood. **(c–f)** Conidiophores and conidia. **(g–i)** Conidiogenous cells with attached conidia. **(j)** Germinating conidium. **(k–m)** Conidia. **(n,o)** Colonies on PDA observed from above and below. Scale bars: **(c–f)** = 20 μm, **(g–j)** = 10 μm, **(k–m)** = 5 μm.

***Helicosporium hainanense*** Y.Z. Lu & J.C. Kang, sp. nov. Figure 6.

*Index Fungorum number*: IF 900031; *Facesoffungi number*: FoF 13101.

*Holotype*: GZAAS 22-2006.

*Etymology*: **“***hainanense*” referring to collecting site.

*Saprobic* on decaying woody substrate. **Sexual morph** Undetermined. **Asexual morph** Hyphomycetous, helicosporous. *Colonies* on the substratum superficial, effuse, gregarious, yellow green. *Mycelium* partly immersed, pale brown to brown, septate, branched hyphae, with masses of crowded, glistening conidia. *Conidiophores* 118–182 μm long, 2.5–4 μm wide ($\bar{x}$ = 155 × 3 μm, *n* = 30), macronematous, mononematous, cylindrical, unbranched, straight or slightly flexuous, septate, pale brown to dark brown, smooth-walled. *Conidiogenous cells* holoblastic, mono- to polyblastic, discrete, determinate, rising laterally from the lower portion of the conidiophores as tiny bladder-like protrusions, 2–8.5 μm long, 1.5–3.5 μm diam., each bearing 1–3 tiny conidiogenous loci, hyaline to pale brown, smooth-walled. *Conidia* 11–13 μm diam. and conidial filament 2–3 μm wide ($\bar{x}$ = 12 × 2.5 μm, *n* = 20), 55–60 μm long, solitary, pleurogenous, helicoid, tightly coiled 2^1^/_4_-2^3^/_4_ times, do not become loose in water, tapering toward the rounded ends, indistinctly multi-septate, guttulate, hyaline to yellowish, smooth-walled.

*Culture characteristics*: *Conidia* germinating on water agar and germ tubes produced from conidia within 12 h. *Colonies* growing on PDA, irregular, with a flat surface, edge undulate, reaching 19 mm in 5 weeks at 25°C, brown to dark brown in the PDA medium.

*Material examined*: CHINA, Hainan Province, Changjiang, Baomeiling, on decaying wood in a terrestrial habitat, 15 August 2021, Jian Ma, BM11 (GZAAS 22-2006, holotype; HKAS 125882, isotype), ex-type living culture, GZCC 22-2006.

*GenBank accession numbers*: OP508730 (ITS), OP508770 (LSU), OP698070 (RPB2), and OP698081 (TEF1α).

Notes: Phylogenetically, *Helicosporium hainanense* shares a sister relationship to *H. flavisporum* and *H. vesicarium* with high statistical support (100% ML/1.00 PP, Fig. 2), and can be considered as a distinct species. Morphologically, *H. hainanense* differs from *H. flavisporum* by its wider and shorter conidial filaments (2–3 μm wide, 55–60 μm long *vs*. 1–2 μm wide, 100–110 μm long), and from *H. vesicarium* by its longer conidiophores (118–182 μm *vs*. 65–120 μm) and smaller conidial diameter (11–13 μm *vs*. 13–18 μm) (Lu et al., 2018b).

***Helicosporium sexuale*** Boonmee, Promputtha & K.D. Hyde, Fungal Diversity 111: 124 (2021), Figure 7.

**Figure 7:**
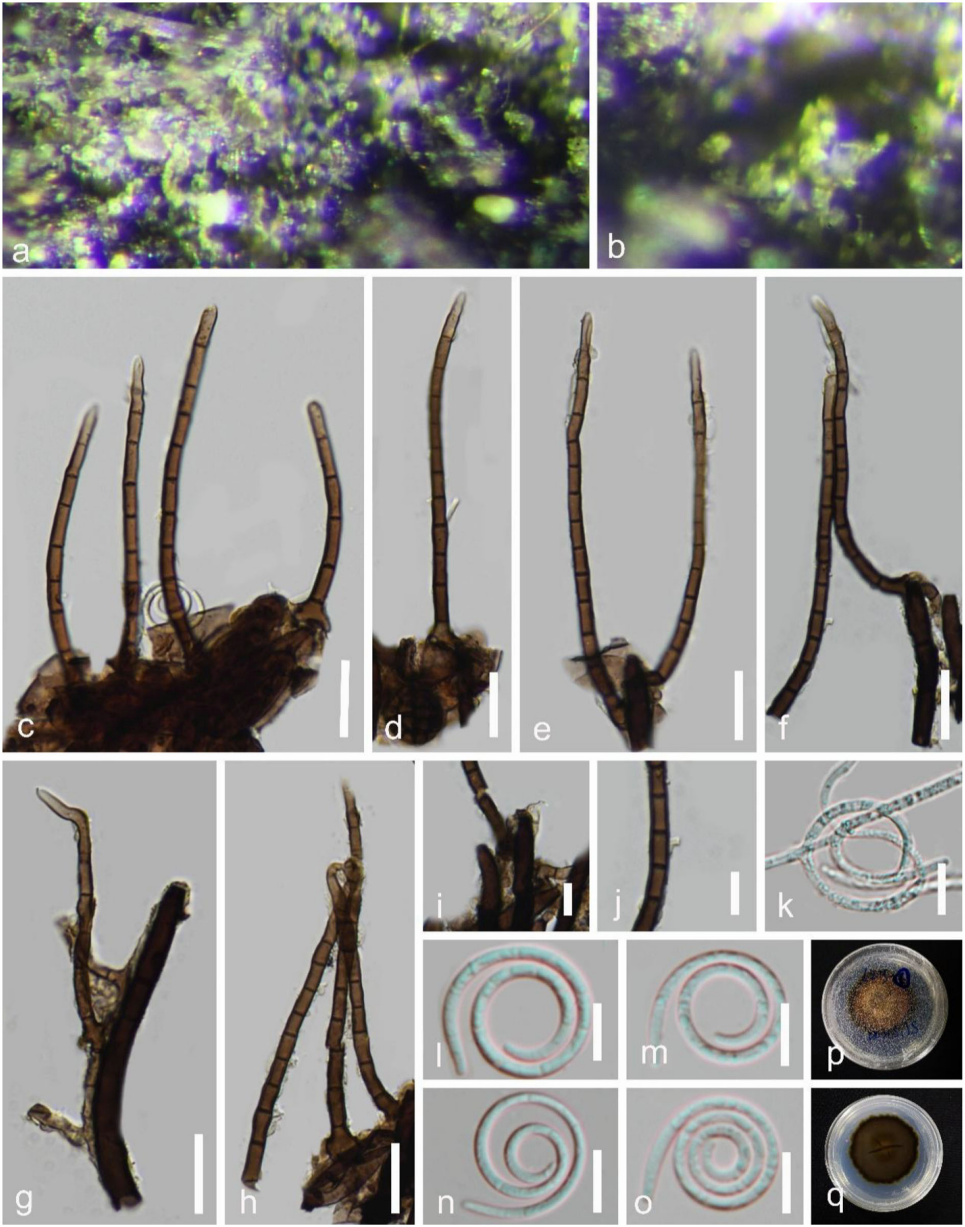
*Helicosporium sexuale* (GZAAS 22-2007). **(a,b)** Colony on decaying wood. **(c–h)** Conidiophores. **(i,j)** Conidiogenous cells. **(k)** Germinating conidium. **(l–o)** Conidia. **(p,q)** Colonies on PDA observed from above and below. Scale bars: **(c–h)** = 20 μm, **(i–o)** = 10 μm.

*Index Fungorum number*: IF 558542; *Facesoffungi number*: FoF 09194.

*Holotype*: MFLU 21-0104.

*Saprobic* on decaying wood in a freshwater stream. **Sexual morph** see Boonmee et al. (2021). **Asexual morph** Hyphomycetous, helicosporous. *Colonies* on the substratum superficial, effuse, gregarious, yellow green. *Mycelium* partly immersed, partly superficial, brown to dark brown, septate, branched hyphae, with masses of crowded, glistening conidia. *Conidiophores* 60–129 μm long, 3.5–6 μm wide ($\bar{x}$ = 98 × 4.5 μm, *n* = 30), macronematous, mononematous, erect, setiferous, cylindrical, septate, brown to dark brown, smooth-walled. *Conidiogenous cells* holoblastic, monoblastic, discrete, determinate, denticulate, rising laterally from the lower parts of conidiophores as tiny tooth-like protrusions, hyaline to pale brown, smooth-walled. *Conidia* 11–20 μm diam. and conidial filament 1–2 μm wide ($\bar{x}$ = 14.5 × 1.5 μm, *n* = 20), 68–91 μm long, solitary, pleurogenous, helicoid, coiled 2–3^1^/_3_ times, becoming loosely coiled in water, rounded at tip, guttulate, indistinctly multi-septate, hyaline to pale green, smooth-walled.

*Culture characteristics*: *Conidia* germinating on water agar and germ tubes produced from conidia within 12 h. *Colonies* growing on PDA, circular, with a flat surface, edge undulate, reaching 40 mm in 6 weeks at 25°C, brown to dark brown in the PDA medium.

*Material examined*: CHINA, Guangxi Zhuang Autonomous Region, Liuzhou City, Luzhai County, on submerged decaying wood in a freshwater stream, 4 May 2021, Jian Ma & Yongzhong Lu, LZ15 (GZAAS 22-2007 = HKAS 125866), living cultures, GZCC 22-2007.

*GenBank accession numbers*: OP508731 (ITS), OP508771 (LSU), OP698071 (RPB2), and OP698082 (TEF1α).

Notes: In this study, a new helicosporous hyphomycete (GZCC 22-2007) was phylogenetically grouped with *Helicosporium sexuale* (MFLUCC 16-1244) and did not show much divergence (Figure 2). We compared their DNA sequences and found that only 5 bp nucleotide differences between them in TEF1α sequence data, whereas their ITS, LSU, and RPB2 sequence data were identical. Therefore, we identify the new isolate GZCC 22-2007 as *H. sexuale. Helicosporium sexuale* was described as only a sexual morph (Boonmee et al., 2021). Its asexual morph is reported in this study for the first time. This is also the first record of *H. sexuale* in a freshwater habitat in China.

**Figure 8:**
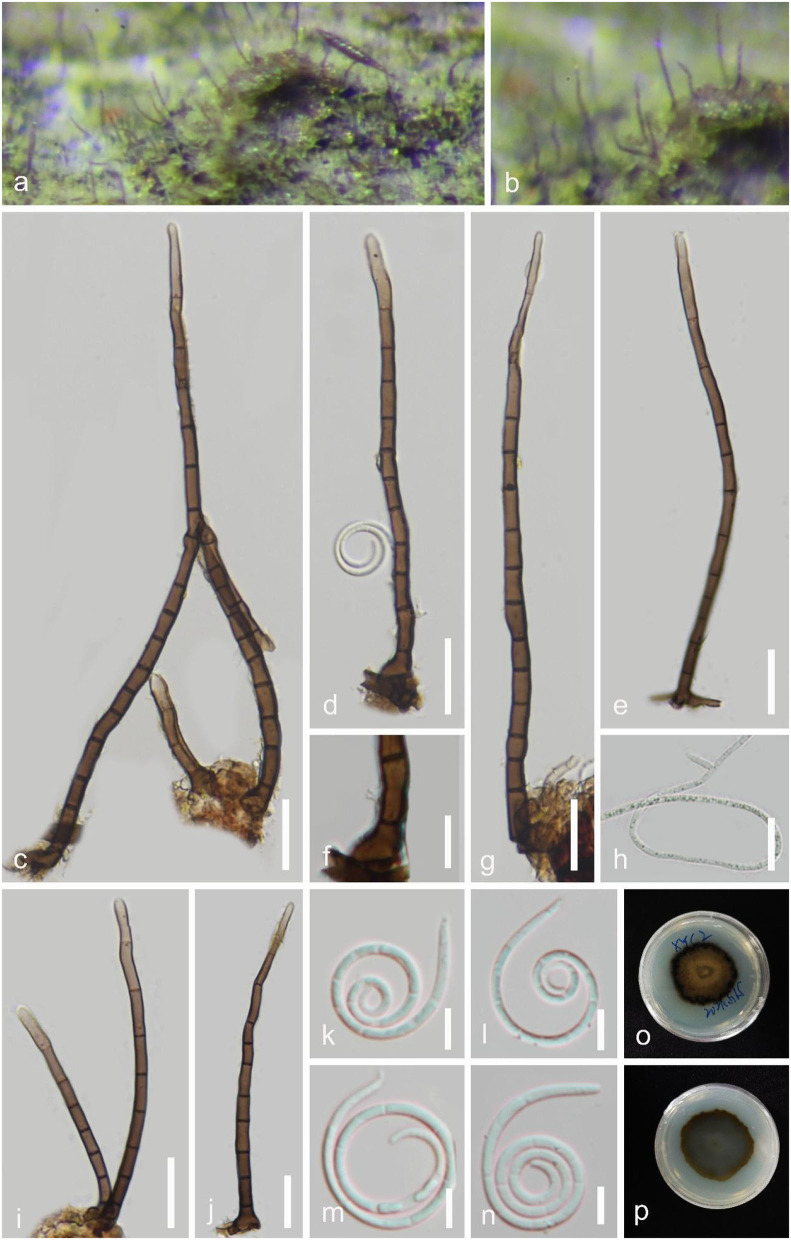
*Helicosporium viridisporum* (GZAAS 22-2008, holotype). **(a,b)** Colony on decaying wood. **(c–e,g,i,j)** Conidiophores and conidia. **(f)** Conidiogenous cells. **(h)** Germinating conidium. **(k–n)** Conidia. **(o,p)** Colonies on PDA observed from above and below. Scale bars: **(c–f,i,j)** = 20 μm, **(g,h)** = 10 μm, **(k–n)** = 5 μm.

***Helicosporium viridisporum*** Y.Z. Lu & J.C. Kang, sp. nov. Figure 8.

*Index Fungorum number*: IF 900030; *Facesoffungi number*: FoF 13102.

*Holotype*: GZAAS 22-2008.

*Etymology*: **“***viridisporum*” referring to the bright lime green conidia in a natural woody substrate.

*Saprobic* on decaying wood in a freshwater stream. **Sexual morph** Undetermined. **Asexual morph** Hyphomycetous, helicosporous. *Colonies* on the substratum superficial, effuse, gregarious, bright lime green. *Mycelium* partly immersed, brown to dark brown, septate, branched hyphae, with masses of crowded, glistening conidia. *Conidiophores* 80–206 μm long, 3–7 μm wide ($\bar{x}$ = 146 × 5 μm, *n* = 30), macronematous, mononematous, erect, setiferous, cylindrical, septate, brown to dark brown, smooth-walled. *Conidiogenous cells* holoblastic, polyblastic, discrete, determinate, denticulate, rising laterally from the lower parts of conidiophores as tiny tooth-like protrusions, hyaline to pale brown, smooth-walled. *Conidia* solitary, 12–14 μm diam. and conidial filament 1–2 μm wide ($\bar{x}$ = 13 × 1.5 μm, *n* = 30), 75–97 μm long, pleurogenous, helicoid, tightly coiled 2–3^1^/_3_ times, becoming loosely coiled in water, rounded at tip, guttulate, indistinctly multi-septate, hyaline to pale green, smooth-walled.

*Culture characteristics*: *Conidia* germinating on water agar and germ tubes produced from conidia within 12 h. *Colonies* growing on PDA, circular, with a flat surface, edge undulate, reaching 40 mm in 5 weeks at 25°C, brown to dark brown in the PDA medium.

*Material examined*: CHINA, Guangxi Zhuang Autonomous Region, Hechi City, Xiayi Village, on submerged decaying wood in a freshwater stream, 3 May 2021, Jian Ma, XYC2 (GZAAS 22-2008, holotype; HKAS 125857, isotype), ex-type living culture, GZCC 22-2008.

*GenBank accession numbers*: OP508736 (ITS), OP508776 (LSU), OP698076 (RPB2), and OP698087 (TEF1α).

Notes: *Helicosporium viridisporum* is a typical *Helicosporium* species according to the redefined generic concept of *Helicosporium* by Lu et al. (2018b). Its colonies on natural woody substratum are bright lime green. *H. viridisporum* shares a sister relationship to *H. sexuale* and can be distinguished by its longer conidiophores (80–206 μm vs. 60–129 μm). The multi-gene phylogenetic analysis supports it as a new species.

**Figure 9:**
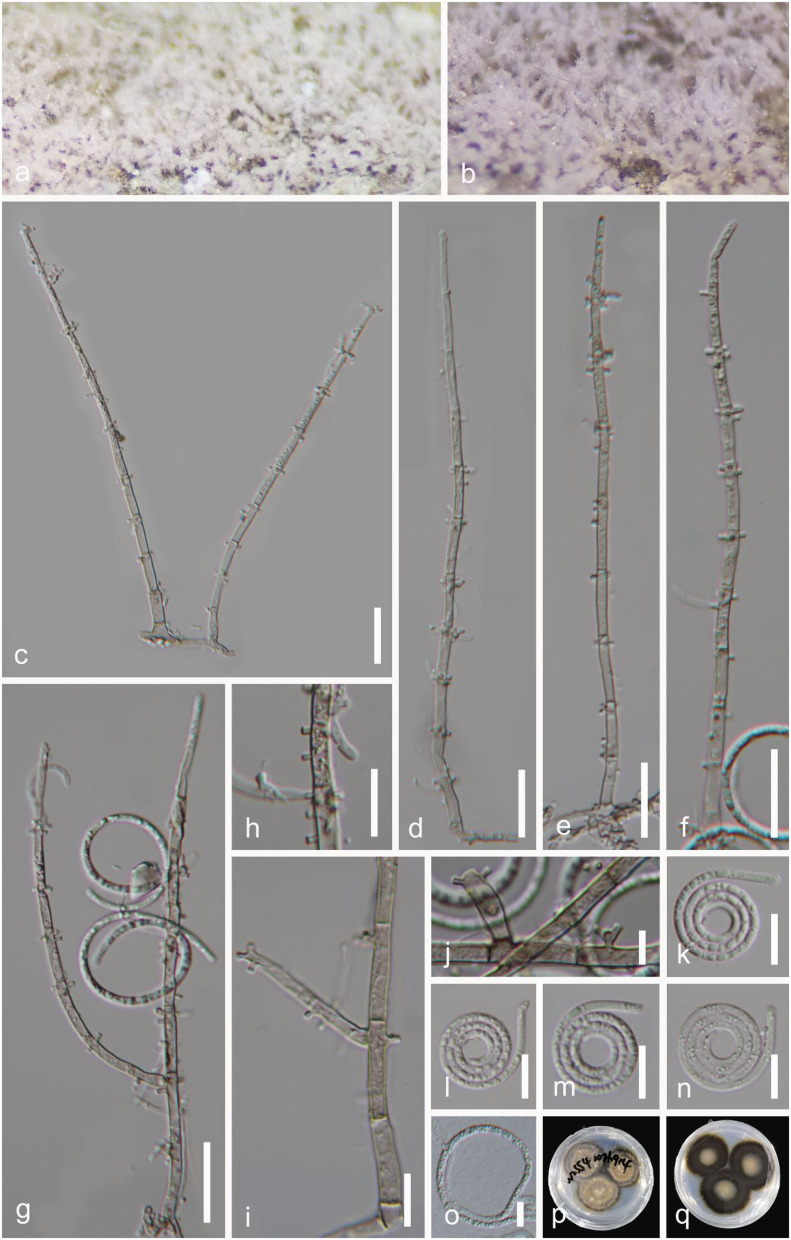
*Neohelicomyces hainanensis* (GZAAS 22-2009, holotype). **(a,b)** Colony on decaying wood. **(c–g)** Conidiophores and conidia. **(h–j)** Conidiogenous cells. **(k–n)** Conidia. **(o)** Germinating conidium. **(p,q)** Colonies on PDA observed from above and below. Scale bars: **(c–g)** = 20 μm, **(h,i,k–n)** = 10 μm, **(j)** = 5 μm.

***Neohelicomyces hainanensis*** Y.Z. Lu & J.C. Kang, sp. nov. Figure 9.

*Index Fungorum number*: IF 900029; *Facesoffungi number*: FoF 13103.

*Holotype*: GZAAS 22-2009.

*Etymology*: **“***hainanensis*” referring to the collection site.

*Saprobic* on decaying wood. **Sexual morph**: Undetermined. **Asexual morph** Hyphomycetous, helicosporous. *Colonies* on the substratum superficial, effuse, gregarious, white to pink. *Mycelium* partly immersed, hyaline to pale brown, septate, with masses of crowded, glistening conidia. *Conidiophores* 137–197 μm long, 2.5–5 μm wide ($\bar{x}$ = 170 × 4 μm, *n* = 30), macronematous, mononematous, erect, septate, sparsely branched, pale brown, rising directly on the substrate, hyaline to pale brown, smooth-walled. *Conidiogenous cells* 11–17 × 3–4 μm ($\bar{x}$ = 14 × 3.5 μm, *n* = 30), holoblastic, mono- to polyblastic, integrated, cylindrical, with lateral minute denticles (1–2 μm long, 1–1.5 μm wide). *Conidia* 14–21 μm in diam., 1.5–3 μm wide ($\bar{x}$ = 17 × 2 μm, *n* = 30), conidial filament 82–136 μm long, solitary, acropleurogenous, helicoid, coiled 2^1^/_2_-3^3^/_4_ times, becoming loosely coiled in water, rounded at tip, guttulate, indistinctly multi-septate, hyaline to yellowish, smooth-walled.

*Culture characteristics*: *Conidia* germinating on water agar and germ tubes produced from conidia within 12 h. *Colonies* growing on PDA, circular, with umbonate surface, edge entire, reaching 29 mm in 5 weeks at 25°C, pale brown to brown.

*Material examined*: CHIAN, Hainan Province, Wuzhishan City, Shuimanhe tropical rainforest scenic area in Wuzhishan, on decaying wood in a terrestrial habitat, 24 August 2021, Jian Ma, WZS54 (GZAAS 22-2009, holotype; HKAS 125863, isotype), ex-type living culture, GZCC 22-2009; Ibid., WZS69 (GZAAS 22-2027, paratype), living culture, GZCC 22-2027.

*GenBank accession numbers*: GZCC 22-2009: OP508734 (ITS), OP508774 (LSU), OP698074 (RPB2), and OP698085 (TEF1α); GZCC 22-2027: OP508735 (ITS), OP508775 (LSU), OP698075 (RPB2), and OP698086 (TEF1α).

Notes: The conidiophores and conidial features of *Neohelicomyces hainanensis* are morphologically similar to those of *N. hyalosporus* but it can be distinguished from *N. hyalosporus* by its shorter conidiophores (137–197 μm vs. 210–290 μm) (Lu et al., 2018b). Its colonies change from white to pink on a natural woody substrate; a feature that other species of the genus do not have. Phylogenetically, *N. hainanensis* shares a sister relationship to *N. pallidus* with high statistical support (97 MLBS/0.99 PP), and the phylogenetic analysis results support it as a distinct species (Figure 2).

## Discussion

The difficulty in the taxonomic study of helicosporous hyphomycete species is that their morphological characteristics are very similar; it is difficult to distinguish them only by morphological comparison (Linder, 1929; Pirozynski, 1972; Goos, 1985, 1986, 1989; Zhao et al., 2007; Kuo and Goh, 2018; Lu et al., 2018a,b; Hsieh et al., 2021; Tian et al., 2022). Therefore, polygenic phylogenetic analysis is required to accurately identify them. However, previous studies have mainly focused on the description of morphological characteristics; most of them without obtaining strains and DNA molecular data (Linder, 1929; Pirozynski, 1972; Goos, 1985, 1986, 1989; Zhao et al., 2007). What makes things more complicated is that standards for species identification are not uniform, which creates confusion in this taxonomic system. Some helicosporous fungi have been transferred several times. For example, Moore (1957) treated *Drepanospora pannosa* as *Helicosporium pannosum*; Matsushima (1975) classified *Drepanospora pannosa, Helicosporium linderi, Helicosporium nematosporum*, and *Helicosporium serpentinum* under *Helicosporium pannosum*; Goos (1989) treated them as *Drepanospora pannosum*; Zhao et al. (2007) treated all of them and *Helicosporium gigasporum* as *Helicosporium pannosum*. The reason the authors reassessed the taxonomic status of these species is that there were some differences in the morphological characteristics of the conidiophores, conidiogenous cells, and conidia; the authors used different taxonomic principles to identify these species (Moore, 1957; Matsushima, 1975; Goos, 1989; Zhao et al., 2007). In our previous study, we paid attention to the confusion regarding the classification of helicosporous hyphomycete, analyzed the existing problems, and proposed ideas to solve the problems (Lu et al., 2018b). Lu et al. (2018b) provided several examples to show that the morphological characteristics of conidiophores, conidiogenous cells, and conidia, including their color and size, are very important influencing factors that cannot be ignored in distinguishing helicosporous fungi. The key to solve this taxonomic system problem is to obtain more species resources such as molecular data and morphological characteristics, for both newly collected specimens and published specimens with incomplete morphological features. Specimens observed in previously published literature that have molecular data but lack morphological characteristics, and are well preserved, can be borrowed for further morphological research.

In addition, different fungal species with similar morphologies produced distinctly characteristic secondary metabolites. For example, the stromata and ascospores of *Annulohypoxylon urceolatum* were morphologically similar to those in *A. leptascum*. However, they could be distinguished by their unique stromatal HPLC profiles, in which *A. urceolatum* produced the sole main metabolite *viz*. urceoline, while *A. leptascum* produced large quantities of truncatone A and C (Kuhnert et al., 2017). *Annulohypoxylon yungensis* was morphologically similar to *A. truncatum*, but the former produced BNT (1,1′-binaphthalene-4,4′-5,5′-tetrol), whereas the latter produced truncaquenone A and B in large quantities as well as trace truncatone A (Surup et al., 2016; Kuhnert et al., 2017). Kuhnert et al. (2017) provided a good example, using chemotaxonomy to evaluate the taxonomic systems of fungi with similar morphologies. This may be a new way to solve the problem of the taxonomy of helicosporous hyphomycetes by using evidence from chemotaxonomic data together with phylogenetic and morphological data.

In this study, we obtained 11 helicosporous fungal specimens and cultures and introduced four new species and three new records of helicosporous hyphomycetes based on morphological and phylogenetic evidence. We are also carrying out studies on the secondary metabolites of these fungi, and hope to find the characteristic compounds of each genus and solve the classification problem of helicosporous fungi with evidence from chemotaxonomic data in future.

## Data availability statement

The datasets presented in this study can be found in online repositories. The names of the repository/repositories and accession number(s) can be found in the article/supplementary material.

## Author contributions

Y-ZL and JM conducted the experiments, analyzed the data, and wrote the article. J-CK planned the experiments. X-JX and Y-PX analyzed the data. JM and X-JX conducted the experiments. L-JZ and J-CK revised the article. Y-ZL and J-CK funded the experiments. All authors revised and agreed to the published version of the article.

## Funding

This work was funded by the National Natural Science Foundation of China (NSFC 31900020, 32170019, and 31670027), the Science and Technology Foundation of Guizhou Province ([2020]1Y058), the China Post-doctoral Science Foundation Project (2020M683657XB), and the Guizhou Province high-level talent innovation and entrepreneurship merit funding project (No. 202104).

## Conflict of interest

The authors declare that the research was conducted in the absence of any commercial or financial relationships that could be construed as a potential conflict of interest.

## Publisher's note

All claims expressed in this article are solely those of the authors and do not necessarily represent those of their affiliated organizations, or those of the publisher, the editors and the reviewers. Any product that may be evaluated in this article, or claim that may be made by its manufacturer, is not guaranteed or endorsed by the publisher.
